# Serum proteome modulations upon treatment provides biological insight on response to treatment in relapsed mantle cell lymphoma

**DOI:** 10.1002/cnr2.1524

**Published:** 2021-07-28

**Authors:** Lavanya Lokhande, Venera Kuci Emruli, Christian Winther Eskelund, Arne Kolstad, Martin Hutchings, Riikka Räty, Carsten Utoft Niemann, Kirsten Grønbæk, Mats Jerkeman, Sara Ek

**Affiliations:** ^1^ Department of Immunotechnology Lund University Lund Sweden; ^2^ Department of Haematology, Rigshospitalet Copenhagen University Hospital Copenhagen Denmark; ^3^ Biotech Research and Innovation Centre BRIC University of Copenhagen Copenhagen Denmark; ^4^ Oslo University Hospital Oslo Norway; ^5^ Department of Hematology Helsinki University Central Hospital Helsinki Finland; ^6^ Department of Clinical Medicine University of Copenhagen Copenhagen Denmark; ^7^ The Danish Stem Cell Center (Danstem), Faculty of Health and Medical Sciences University of Copenhagen Copenhagen Denmark; ^8^ Department of Oncology Lund University Lund Sweden

**Keywords:** biomarker discovery, mantle cell lymphoma (MCL), serum proteins

## Abstract

**Background:**

The possibility to monitor patient's serum proteome during treatment can provide deepened understanding of the biology associated with response to specific drugs. Non‐invasive serum sampling provides an opportunity for sustainable repetitive sampling of patients, which allows for more frequent evaluation of the biological response and enhanced flexibility in treatment selection in contrast to tissue biopsies.

**Aim:**

To pin‐point biologically relevant changes in pre‐ and on‐treatment serum proteome samples in relapsed mantle cell lymphoma (MCL) patients, leading to insight into mechanisms behind response to treatment in sub‐groups of patients.

**Methods:**

Pre‐ and on‐treatment serum samples from relapsed MCL patients treated with a triple combination therapy of rituximab, ibrutinib and lenalidomide were available for the study, together with detailed clinicopathological information. A microarray technology targeting 158 serum proteins using 371 antibody‐fragments was used to compare the serum proteome at the two time‐points.

**Results:**

Proteins modulated by the treatment were shown to be associated to a MCL sub‐group with *ATM/TP53* alterations, which emphasizes the importance of treatment stratification. Absolute values of serum protein levels in on‐treatment samples were highly variable and showed no correlation to outcome. To circumvent the challenge of variability in absolute serum protein levels, the velocity of change of individual serum proteins was used to identify proteins associated with clinical response. Increased values of TGF‐β1, CD40 and complement component 4 comparing pre‐ and on‐treatment samples were associated with remaining minimal residual disease (MRD) and increased BTK was associated with short progression‐free survival (PFS).

**Conclusion:**

We show that the genetic sub‐type of MCL affects the biological response to treatment in serum and that the change in defined serum proteins reveals the biology associated with clinical response.

## 1 INTRODUCTION

Mantle cell lymphoma (MCL) remains a subtype of non‐Hodgkin's lymphoma (NHL) with high relapse rates and poor prognosis.^1^ Over the last few decades, the increase in understanding of the molecular pathogenesis of this disease has enabled development of new therapeutic regimens with emphasis on anti‐CD20 monoclonal antibody immunotherapies in conjugation with prevalent chemotherapeutic strategies.^2^ This has improved the overall survival and lowered the progression rate. However, further improvement in combinatorial therapeutics is needed for better outcome, particularly in patients with relapsed/refractory (R/R) MCL.

To aid in this process, improved insight of how the current therapies modulate the biology is essential. Using serum, it is possible to analyze the effect on the systemic immune profile by various treatments, as samples across multiple time‐points easily can be collected. Changes in serum proteome can then be used to gain clinically relevant insights.^3^ ^,^ ^4^ This is especially important for aggressive systemic diseases like MCL where repeated invasive sampling through tissues biopsies is not feasible.

Serum proteins have proven to be extremely valuable to gain diagnostic and prognostic information in various cancer types with several biomarkers being clinically implemented; either as individual markers (e.g., prostate‐specific antigen [PSA] for prostate cancer diagnosis and prognosis) or combined signatures (e.g., OVA1 five protein panel for ovarian cancer).^5^, ^6^ The focus of serum analysis in most published studies is on pre‐treatment cohorts for biomarker discovery in relation to diagnosis and treatment outcome predictions. As serum proteins are in a state of constant flux, especially when the patient's treatment status changes, there is additional information to be collected in on‐treatment sampling for improved understanding of disease progression. Few studies have analyzed the on‐treatment modulation of serum proteins for B‐cell lymphomas before and during treatment,^7^, ^8^, ^9^ and none for MCL.

In the present study, we took benefit of the combined collection of pre‐ and on‐treatment serum samples and clinicopathological information from the Nordic MCL6‐Philemon clinical trial for relapsed/refractory (R/R) MCL.^10^, ^11^ The trial includes immunomodulatory treatment with lenalidomide, BTKi ibrutinib and anti‐CD20 antibody rituximab. In a previous study, a prognostic signature (RIS; relapsed immune signature) based on 11 serum proteins correlated to overall survival was developed using the pre‐treatment samples.^12^ Clinical and biological information was integrated and the MCL international index/relapsed immune signature (MIPI_ris_) was developed and showed improved stratification of patients into three risk groups.^12^ A separate previous study has also investigated the most common genetic alterations, including *ATM* and *TP53* and their correlation to clinical outcome in MCL.^13^ In this follow‐up study, the IMMray™ protein microarray technology was used to investigate changes in serum proteome pre‐ and on‐treatment.

The aim of the study is to pin‐point changes in pre‐ and on‐treatment serum proteome samples, leading to improved biological insight of relapsed MCL and response to treatment.

## 2 METHODS

### 2.1 Patient samples and information

**FIGURE 1:**
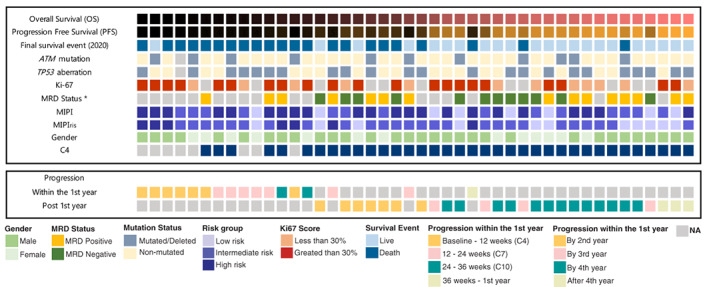
Mapping of the most important outcome and clinicopathological parameters including OS, PFS, survival event at latest follow‐up, *ATM* and *TP53* aberrations, Ki‐67, MRD, MIPI, MIPI_ris_ and gender (upper panel). Patients are sorted according to OS (low (black) to high (pink)). Likewise, PFS is also represented in a range (low(black) to high (orange). NA (gray) represents missing data points. The minimal residual disease (MRD) status (*) was measured at 6 months after treatment initiation using bone marrow. For each patient, the time to progression is shown as a color code (lower panel)

Serum samples were collected from patients included in R/R MCL6‐Philemon clinical trial (NCT02460276) conducted by the Nordic Lymphoma group (patients enrolled 2015–2016, with last follow‐up date in December 2019). The patients received induction treatment with lenalidomide, ibrutinib and rituximab (12 cycles, 28 days each).^10^ This was followed by a maintenance phase of rituximab and ibrutinib. Samples were collected pre‐treatment (Baseline samples, *n* = 44), and on‐treatment (at cycle 4, after 12 weeks of treatment, *n* = 36) which is the second time‐point used for serum analyses in the present study.^11^ Samples from eight patients were lacking at the second time point. Five samples were lacking due to death of the patients, whereas three serum samples were not available and hence, not evaluated in the microarray (Figure 1). Serum samples were stored at −80°C until the day of the experiment. The overall patient characteristics are shown in Figure 1 and in Table S1.

The patient information and material collected from this trial was previously used to assess the treatment regimen efficacy^10^, ^11^ and to identify gene mutations or deletions.^11^ Serum analysis on the pre‐treatment samples has been published, which led to the formation of a prognostic serum protein signature strengthening the established MIPI, and the development of the MIPI_ris_.^12^ The current study is a follow‐up of the previous serum analysis to study the effect of treatment on the serum proteome, and which biological information can be retrieved on response to therapy in the relapsed setting. The experimental setup is identical to the published study and explained in detail in Lokhande et al, 2020.^12^

For this cohort of patients (*n* = 44), the median follow‐up time is around 5 years, with a median survival of 40.3 months and a median progression free survival of 17.6 months. Additionally, 25% of patients (*n* = 11) had *ATM* mutation and 52% (*n* = 23) of patients had *TP53* aberrations (mutations or deletions; Figure 1). In total, 15.9% (*n* = 7) of patients had aberrations in both *ATM* and *TP53* (Figure 1). The mutation status was analyzed from DNA extracted from bone marrow samples, using a custom‐designed multiplex Ion Ampliseq panel (Ampliseq designer, Thermo Fischer Scientific, Waltham, MA) described in Eskelund et al.^13^ As reported previously,^11^ no significant correlation between survival and *TP53* aberrations was seen, in contrast to studies where patients have been treated with chemotherapy‐based regimens in which *TP53* mutations is a poor prognostic marker.^14^, ^15^ To determine minimal residual disease (MRD), PCR amplification of immunoglobulin heavy chain genes (IGH) and CCND1‐IGH t(11;14) translocations were assessed from bone marrow derived DNA, as previously described.^11^

### 2.2 Serum protein microarray and data pre‐processing

The serum samples (*n* = 80) were analyzed using an antibody microarray platform with 371 single chain antibody fragments targeting 158 immunoregulatory serum proteins. The antibody‐target list can be found in Table S2 and the experimental protocol has been explained in detail in Lokhande et al.^12^ The generated dataset was quality controlled, followed by ComBat normalization (using surrogate variable analysis [SVA] package, www.r-project.org) to remove batch effects.^12^

### 2.3 Data analysis

Two‐group comparison and p‐value filtering (*α*‐cutoff = .05) using Qlucore (Qlucore, Lund, Sweden) was used to identify differentially regulated proteins in paired samples (B vs. C4). GraphPad prism 9 (GraphPad software, San Diego, CA) was used to create the box‐and‐whisker plots and to perform two‐tailed paired t‐test for assessing the intensity distribution before and after‐treatment.

The difference was defined as the log ratio of signal intensity of paired samples (*n* = 36), hereafter named as velocity of change or δ_C4,_ representing the rate of change in serum expression comparing pre and on‐treatment expression profiles. Thus, δ_C4_ < 0 indicated higher expression and δ_C4_ > 0 indicated lower expression in the on‐treatment samples in comparison to the pre‐treatment samples. Qlucore omics explorer (Version 3.6, Qlucore, Lund, Sweden) was used for heatmap, volcano plot and feature reduction using group comparison with a *p*‐value cut‐off of .05. To identify key biomarkers in relation to time‐to‐progression (TTP), patients were grouped based on PFS (Figure S1(A)). Early progression and late progression were classified as patients that progressed within the first year (*n* = 17) and during/post fourth year (*n* = 16), respectively (Figure S1).

The remaining graphs and other analysis (Kaplan–Meier curve, time‐series graph, log‐rank tests) were created using R and R studio (www.r-project.org). For functional analysis of proteins, pathway analysis was performed using STRING: The Search Tool for the Retrieval of Interacting Genes/Proteins (http://stringdb.org). The entire analysis workflow for the study is given in Figure S2.

## 3 RESULTS

### 3.1 Differentially expressed biomarkers between pre‐ and on‐treatment samples are associated with specific genetic subtypes

**FIGURE 2:**
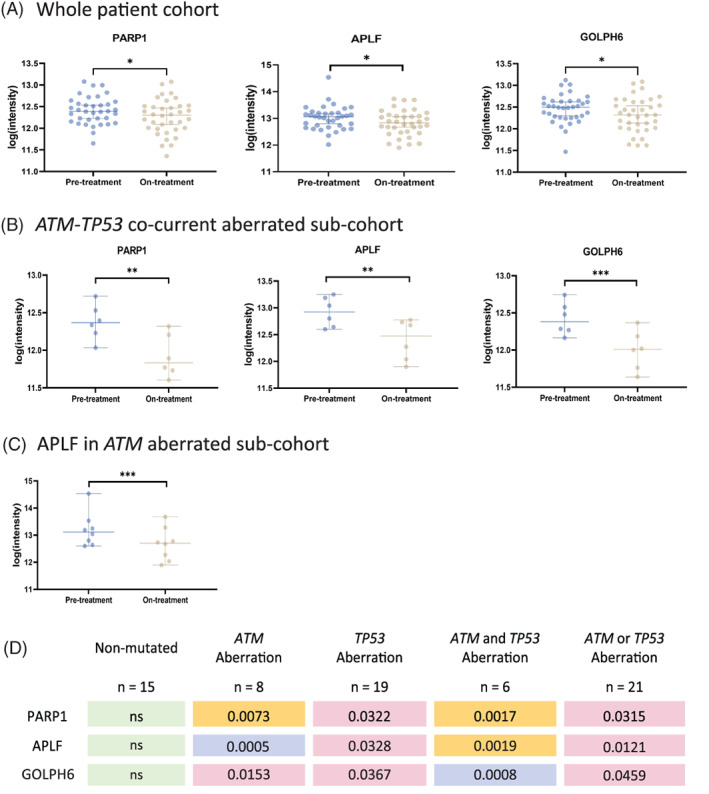
Serum expression levels for each of the three proteins differentially expressed comparing pre‐ and on‐treatment samples. (A) Three proteins PARP1, APLF and GOLPH6 were identified using Qlucore™ after variance and *p*‐value (*α* < .05) filtering using two group comparison. Differential serum expression levels between pre‐ and on‐treatment patients with (B) *ATM‐TP53* co‐current aberrations for PARP1, APLF and GOLPH6 respectively and (C) APLF profile for *ATM* mutated patients. (D) Table with the p‐value (paired analysis) of the three serum proteins in the different genetic sub‐types of MCL. Boxes are colored in accordance with p‐value (green: non‐significant [ns], pink: *p* < .05, yellow: *p* < .01 and blue: *p* < .001). Based on the overall analysis, *ATM* aberrations seem to be more significant, but co‐current samples overall contribute the most to the expression profile. *p < .05, **p < .01, ***p < .001

With the aim of increasing biological understanding of disease progression, we performed comparative analysis of serum proteins in pre‐ and on‐treatment samples to find differentially regulated proteins. Feature reduction of the entire antibody panel in conjunction with paired (*n* = 35) two‐group comparison (Qlucore), yielded three statistically significantly (*p* < .05) differentially regulated proteins: Poly (ADP‐Ribose) Polymerase 1 (PARP1), Aprataxin, PNK‐like factor (APLF) and Golgi reassembly‐stacking protein 2 (GOLPH6; Figure 2(A)). The three proteins had reduced expression in the on‐treatment samples. However, this decrease was exclusively contributed by patients with aberrations of *TP53* and/or *ATM* (Figure 2(B)). Co‐current *ATM/TP53* aberrations showed association with decreased expression for both GOLPH6 and PARP1 (*p* ~ .0008 and *p* ~ .0017 respectively) (Figure 2(B),(D)). APLF showed decreased serum expression at C4 with the most significant difference in the *ATM* mutated patients (*p* ~ .0005; Figure 2(C), (D)). The non‐mutated patients did not exhibit any differential profile between pre‐ and on‐treatment samples for the three identified proteins (Figure 2(D)). The association to *ATM* and *TP53* mutations indicates that genomic status plays a role in treatment‐dependent protein modulation.

### 3.2 Evaluation of previously defined RIS‐signature

To evaluate if the previously defined RIS‐signature is stable at on‐treatment, the signature was evaluated using the serum protein levels in on‐treatment samples. We show that the trend with higher expression of the RIS‐proteins in the low‐risk group remains the same (Figure S3). However, when using absolute values and comparing high/low‐risk MIPI_ris_ groups, no analytes were significantly regulated within the MIPI_ris_ subgroups in the on‐treatment samples, while more than 200 analytes (data not shown) were statistically differentially regulated between high/low MIPI_ris_ in pre‐treatment samples. Thus, we concluded that although trends identified in pre‐treatment samples remain the same in on‐treatment samples, the biological variation induced by the treatment causes a major challenge in using absolute values in on‐treatment samples.

### 3.3 Velocity profile (δ_C4_ ) circumvents heterogeneity in protein modulation and pinpoints additional biomarkers associated to MRD and progression

To be able to analyze the patient specific response independent of absolute serum protein values, we exploited a strategy of using the velocity of change between pre‐ and on‐treatment samples. This strategy has successfully been employed previously in a similar dataset obtained by the IMMray™ technology for breast cancer clinical cohort study for prediction of metastasis.^16^

**FIGURE 3:**
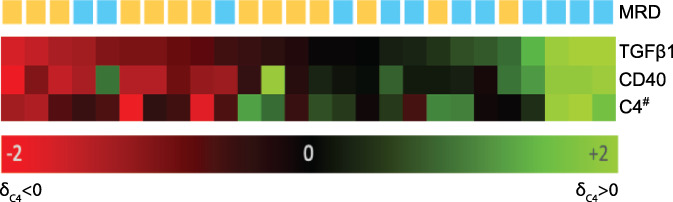
Heatmap showing the expression of the three proteins differentially regulated between patients with MRD (Yellow) and without MRD (Blue) using δ_C4_. MRD was measured using bone marrow samples 6 months after treatment initiation. δ_C4_ < 0 indicates higher expression and δ_C4_ > 0 indicates lower expression in the on‐treatment samples in comparison to the pre‐treatment samples. ^#^Refers to complement component 4

By assessing the panel of proteins using δ_C4_, and associating it to outcome and important clinical parameters, we identified three proteins that were differentially regulated between MRD+ and MRD− patients. TGF‐β1 was the most significant (*p*‐value = .0186) analyte followed by CD40 (*p*‐value = .0275) and complement component 4 (*p*‐value = .048). Overall, MRD+ were associated with increased protein expression in on‐ compared to pre‐treatment samples for the significantly de‐regulated proteins (Figure 3).

**FIGURE 4:**
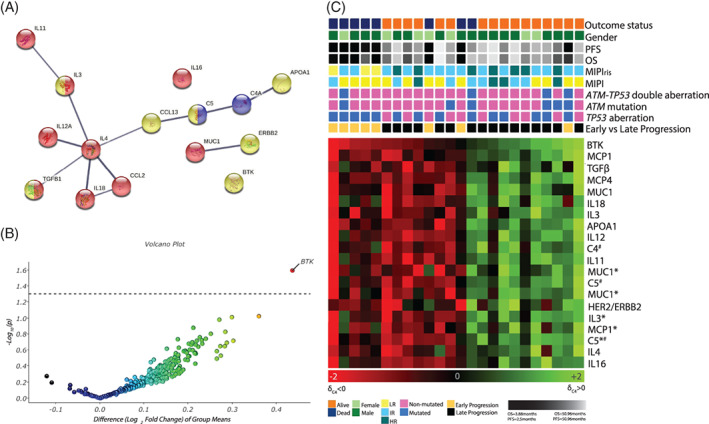
Serum proteins associated with time to progression. (A) Pathway analysis of the top 20 serum proteins differentially modulated (δ_C4_) comparing patients with early and late progression. The identified proteins are broadly associated with interleukin signaling (red), complement cascade (blue) or in signal transduction (yellow). (B) Volcano plot identifying BTK to be significantly (*p*‐value cutoff = .05) overexpressed (comparing on‐ and pre‐treatment samples) in patients with early progression. (C) Heatmap of the top 20 serum proteins sorted in accordance with decreasing p‐value. The heatmap shows the formation of two major clusters based on the protein expression profile of the velocity of change. Various clinical parameters including mutational status, progression and survival have been mapped to the cluster distribution. δ_C4_ < 0 indicates higher expression and δ_C4_ > 0 indicates lower expression in the on‐treatment samples in comparison to the pre‐treatment samples. *Represents protein identified by second antibody clone. ^#^C4/C5 represents complement component 4/5, respectively

To identify key biomarkers in relation to TTP, patients were categorically classified as early and late progression as defined in the Methods section. Using δ_C4_ and the two‐group comparison in Qlucore™, we identified the top 20 differentially regulated serum proteins comparing early and later progression. These proteins mainly consisted of inflammatory cytokines, chemokines and proteins belonging to the complement cascade and can be directly correlated to the immunomodulatory treatment (Figure 4(A)). Among the top 20 proteins, BTK (*p* = .026) was significantly modulated between the early and late progression (Figure 4(B) and Figure S4). Heatmap visualization led to the formation of two clusters of patients with either higher (C4 > B (*n* = 13)) or lower expression of these top 20 proteins comparing pre‐ and on‐treatment samples (C4 < B (n = 11)) (Figure 4(C)). In general, patients with early progression (87.5%) showed increased expression upon treatment for proteins associated with time to progression. In the group with high expression of the top 20 proteins identified by hierarchical clustering, 53% of the patients died during the treatment period, with an average OS of 26.9 months. Contrarily, only 9% of the patients associated with low expression of the top 20 proteins died during the same time period and had an average OS of 43.1 months. The differences in OS were not explained by the frequency of *TP53* mutations (data not shown). Based on the above, we conclude that increasing serum levels of BTK is indicative of continued tumor growth and associated with progression before 12 months. Increasing serum expression of BTK is indicative of poor response to treatment and results in early progression and lower survival (Figure 4 and Figure S4).

## 4 DISCUSSION

A biologically oriented approach to treatment selection and adaptation is a must to improve outcome in relapsed MCL. Here we explored the biology behind treatment response through investigations of pre‐ and on‐treatment samples from a clinical trial including a combination of targeted and immune‐stimulatory agents. By measuring serum proteins, a global view of both the systemic immune response against the tumor as well as the proteins secreted or leaked from the tumor can be assessed, and the non‐invasive sampling allows repetitive collection over time.

Based on our study, we conclude that, (a) MCL subtypes with specific mutations in *TP53* and *ATM* affect the way that the serum proteome changes upon treatment, emphasizing that the response is dependent on specific features of the tumor biology. (b) Using the rate of change/velocity profile, which circumvents the heterogeneity of absolute protein serum levels, serum protein changes associated with early progression and MRD status could be identified.

Comparing pre‐ and on treatment serum samples, we show that three proteins, that is, PARP1, APLF, GOLPH6 were differentially regulated with lower expression after 3 months of treatment and the reduction was primarily seen in patients with alterations in either *TP53* and/or *ATM*. Thus, it seems that MCL patients with a specific mutational spectrum respond more homogenously to the treatment. In particular, PARP1 has been extensively studied in various cancer types,^17^, ^18^, ^19^, ^20^, ^21^ with a focus towards PARP1 inhibitors and synthetic lethality.^22^ The efficacy of these inhibitors in inducing lethality in *ATM* and *ATM‐TP53* double deficient MCL was previously validated using in vitro and in vivo models.^23^, ^24^, ^25^, ^26^, ^27^ Thus, the modulation in PARP1 expression in *ATM‐TP53* aberrated MCLs could be a result of the increased DNA repair burden and targeting PARP1 in these tumors is an interesting clinical option. A potential cause of decreased PARP1 serum expression could be a result of tumor reduction upon treatment, which needs to be further investigated. Ibrutinib that is included in the treatment is known to activate caspase signaling that in the next step causes PARP1 cleavage, a known hallmark of cell death through apoptosis.^28^, ^29^, ^30^, ^31^, ^32^, ^33^ In this study, the antibody‐fragment used targeting PARP1 is directed to the C‐terminal part of the protein and will thus detect either the full protein or the 89 kDa caspase‐cleaved subunit, both including the C‐terminal domain.

APLF and GOLPH6, have not previously been studied in relation Ibrutinib/Lenalidomide or Rituximab. However, APLF functions together with PARP1 in DNA repair mechanisms^34^ and there is also evidence that GOLPH6 is cleaved by caspase‐3 during apoptosis.^35^ Thus, we speculate that the serum expression profile of these three proteins is a result of increased apoptosis of tumor cells.

To improve the biological understanding of treatment effect on serum proteome, as a surrogate marker for local response in the tumor, comparative analysis of the change in serum levels for pre‐ and on‐treatment samples was performed. We showed that the absolute values in on‐treatment samples were highly variable and no correlation to outcome was seen, thus the velocity of change (δ_C4_) in serum protein levels was used to assess changes more robustly. The concept of using the velocity of change has been extensively studied, especially for prostate‐specific antigen (PSA), since the most common problem with PSA has been its variable detection, which leads to overdiagnosis and thus, overtreatment.^36^, ^37^ The use of PSA velocity (PSAV) has been proposed and comprehensively reviewed.^38^, ^39^, ^40^

In our study, we show that the velocity of change (δ_C4_) of TGF‐β1 serum protein along with CD40 and complement component 4 (C4) was different comparing patients with/without MRD at 6 months of treatment. TGF‐β1 has been demonstrated to negatively contribute to persistent MRD positive status in several types of cancer.^41^, ^42^, ^43^ TGF‐β1 can act as a metastatic driver, inducer of EMT transition and impair anti‐tumorigenic response.^44^, ^45^, ^46^ Thus, our results where increased serum TGF‐β1 at 3 months of treatment predicts 6‐months MRD status suggest that targeting and/or using TGF‐β1 to monitor MCL patients is an interesting prognostic tool.

Furthermore, we show that the velocity of change (δ_C4_) of BTK was associated with disease progression. As there is no evidence that BTK is secreted, the serum profile could be a result of the proteins being released from lymphoma cells upon treatment. The possibility to measure BTK in serum as a surrogate marker for tumor burden is an attractive alternative that should be further studied.

In summary, in this conceptually novel study of pre‐ and on‐treatment serum samples in relapsed MCL, we were able to pin‐point novel biological findings with a tentative future clinical implication. We show that the genetic background influences the way that the serum proteome changes upon treatment, confirming that genetic testing is important for treatment selection. We further conclude that using velocity of change between pre‐ and on‐treatment samples enables us to circumvent the heterogeneity in absolute serum levels of biologically interesting markers. Thus, we propose that non‐invasive sampling of serum is useful to monitor patients and provides the possibility to adjust treatment based on biological response. Serum sampling at/or before 12 weeks of treatment should be introduced in future clinical trials to allow similar studies for a wider set of treatment regimens. Based on our data, we conclude that a risk score combining MIPI, key genetic alterations including *TP53* and *ATM*, and velocity of change of key serum markers, is a way forward to reach more personalized MCL treatment plans.

## CONFLICT OF INTEREST

S.E. is an original co‐founder, and stockholder of Immunovia that provided the antibody‐microarrays used in the study. C.U.N. received research funding and/or performed consultancies for Abbvie, AstraZeneca, CSL Behring, Sunesis, Janssen, Roche, Genmab outside this work. Janssen and Celgene provided study drug and financial support for the MCL6‐Philemon trial.

## AUTHOR CONTRIBUTIONS

All authors had full access to the data in the study and take responsibility for the integrity of the data and the accuracy of the data analysis. *Conceptualization*, L.L., M.J., S.E.; *Methodology*, L.L.; *Investigation*, L.L.; *Formal Analysis*, L.L., V.K.E.; *Resources*, C.W.E., A.K., M.H., R.R., C.U.N., K.G., M.J., S.E.; *Writing—Original Draft*, L.L.. S.E.; *Writing—Review & Editing*, L.L.. S.E.; *Visualization*, L.L.; *Supervision*, V.K.E., M.J., S.E.; *Funding Acquisition*, S.E.; Data Curation, L.L., C.W.E., A.K., M.H., R.R., C.U.N, K.G., M.J.

## ETHICAL STATEMENT

The study was approved according to national and international law by the regional ethic committee at Lund University (Lund regional ethic committee, Dnr 2014‐709), and included written informed consent from all participants.

## Supporting information


**Supplementary Table S1** Overall patient distribution
**Supplementary Table S2:** Antibody targets used in the microarray platform. A total of 158 unique proteins across 371 scFv clones.

**Supplementary Figure S1** Number of yearly (A) PFS events and B) deaths during the follow‐up period (2015–2020)

**Supplementary Figure S2** Analysis workflow of the study

**Supplementary Figure S3** Patient dendrogram based on the 11 RIS proteins at pre‐ and on‐treatment samples mapped against the MIPI_ris_ high‐ and low‐risk groups^12^. The distribution shows that the majority (75%) of high‐risk patients remain associated with reduced expression of the 11 RIS proteins in contrast to the low‐risk patients having relatively higher protein expression.

**Supplementary Figure S4** BTK serum expression profile of pre‐treatment and on‐treatment samples grouped by patients with A) early and B) late progression. For missing samples, median value was used as a substitute.

## Data Availability

The data that support the findings of this study are available from the corresponding author upon reasonable request.
