# Supplementary material for: Serum proteome modulations upon treatment provides biological insight on response to treatment in relapsed mantle cell lymphoma
Source: Cancer Rep (Hoboken). 2021 Jul 28;5(7):e1524. doi: 10.1002/cnr2.1524 (PMC9327662; doi:10.1002/cnr2.1524)
Supplement: Supplementary file 1 — Supplementary Table S1 Overall patient distribution Supplementary Table S2: Antibody targets used in the microarray platform. A total of 158 unique proteins across 371 scFv clones. [file CNR2-5-e1524-s005.docx]

**Supporting information**

***Supplementary Table S1:*** Overall patient distribution

**
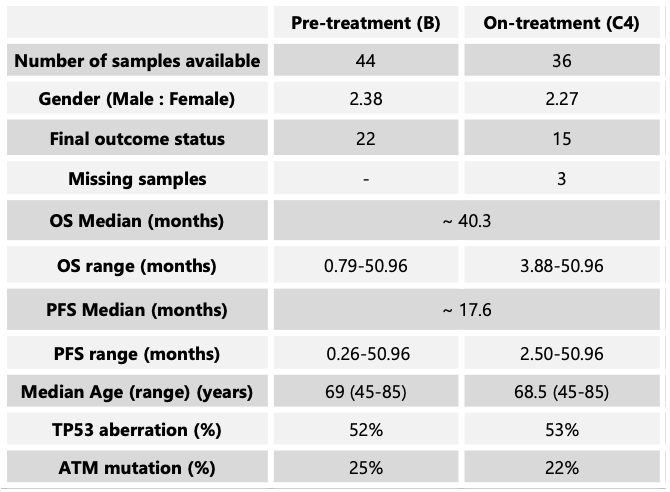
**

***Supplementary Table S2:*** Antibody targets used in the microarray platform. A total of 158 unique proteins across 371 scFv clones.
