# Supplementary figures and images for: Serum proteome modulations upon treatment provides biological insight on response to treatment in relapsed mantle cell lymphoma
Source: Cancer Rep (Hoboken). 2021 Jul 28;5(7):e1524. doi: 10.1002/cnr2.1524 (PMC9327662; doi:10.1002/cnr2.1524)

A

PFS Distribution

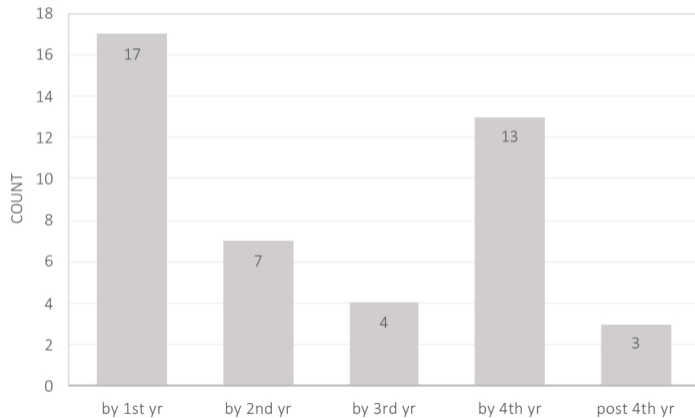

B

Outcome status distribution

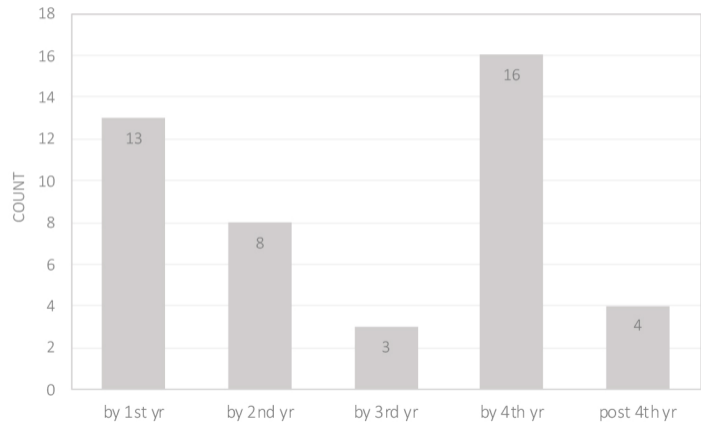

Supplement: Supplementary file 2 — Supplementary Figure S1 Number of yearly (A) PFS events and B) deaths during the follow‐up period (2015–2020) [file CNR2-5-e1524-s003.pdf]

Pre-treatment

On-treatment

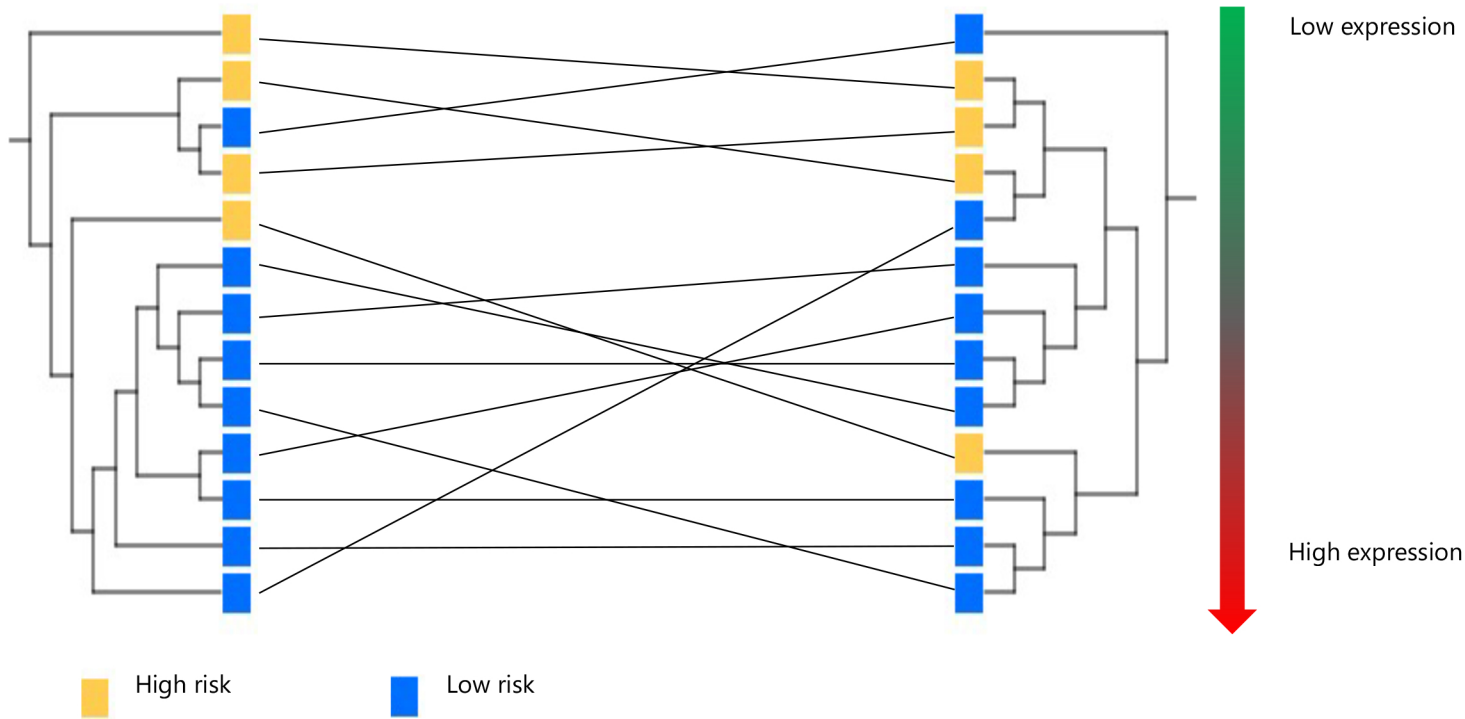

Supplement: Supplementary file 4 — Supplementary Figure S3 Patient dendrogram based on the 11 RIS proteins at pre‐ and on‐treatment samples mapped against the MIPIris high‐ and low‐risk groups12. The distribution shows that the majority (75%) of high‐risk patients remain associated with reduced expression of the 11 RIS proteins in contrast to the low‐risk patients having relatively higher protein expression. [file CNR2-5-e1524-s004.pdf]
