# Supplementary material for: Serum proteome modulations upon treatment provides biological insight on response to treatment in relapsed mantle cell lymphoma
Source: Cancer Rep (Hoboken). 2021 Jul 28;5(7):e1524. doi: 10.1002/cnr2.1524 (PMC9327662; doi:10.1002/cnr2.1524)
Supplement: Supplementary file 3 — Supplementary Figure S2 Analysis workflow of the study [file CNR2-5-e1524-s001.pdf]

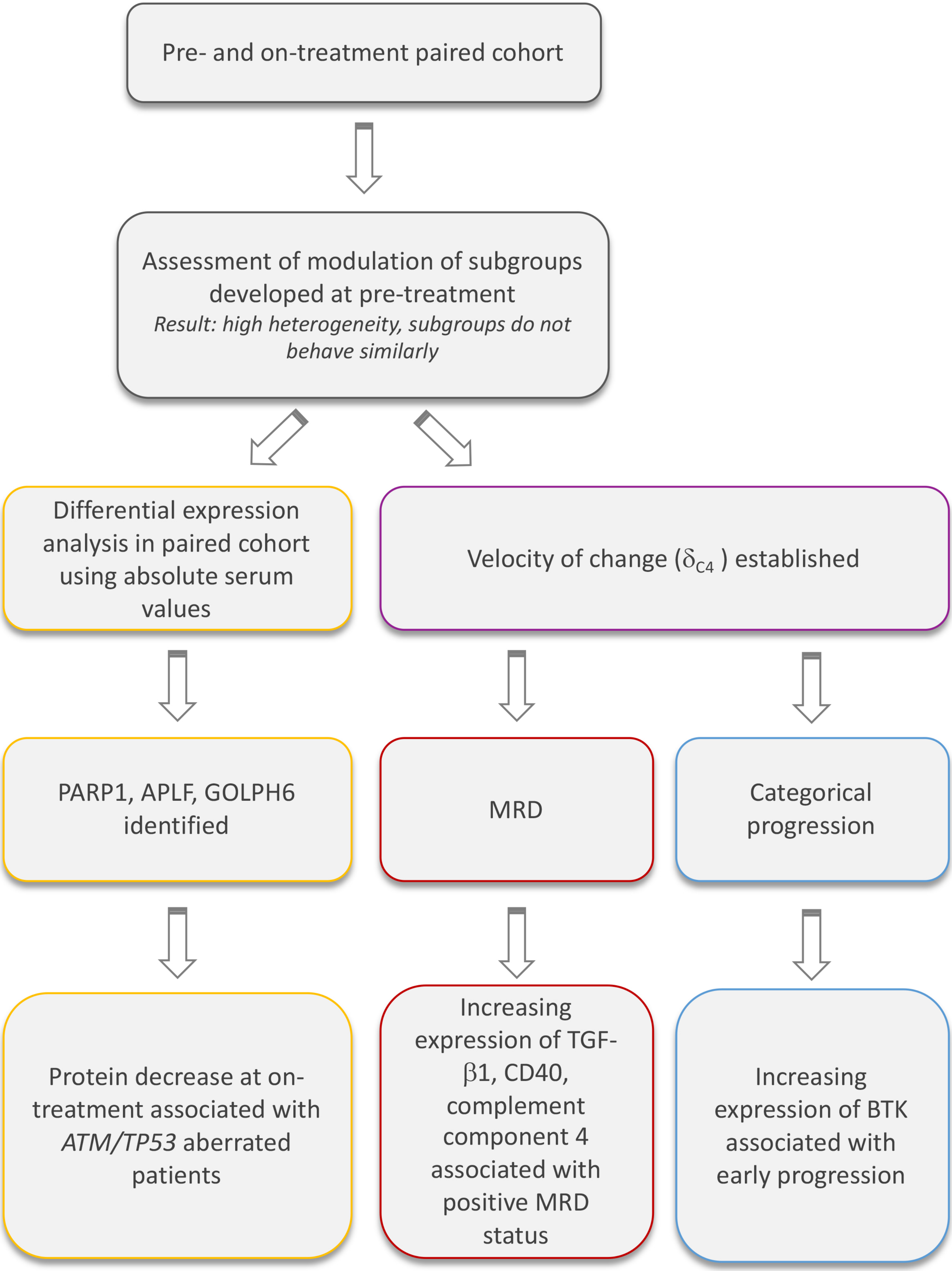
Pre- and on-treatment paired cohort
Assessment of modulation of subgroups developed at pre-treatment
Result: high heterogeneity, subgroups do not behave similarly
Differential expression analysis in paired cohort using absolute serum values
Velocity of change (δ_{C4}) established
PARP1, APLF, GOLPH6 identified
MRD
Categorical progression
Protein decrease at on-treatment associated with *ATM/TP53* aberrated patients
Increasing expression of TGF-β1, CD40, complement component 4 associated with positive MRD status
Increasing expression of BTK associated with early progression
