# Supplementary material for: Serum proteome modulations upon treatment provides biological insight on response to treatment in relapsed mantle cell lymphoma
Source: Cancer Rep (Hoboken). 2021 Jul 28;5(7):e1524. doi: 10.1002/cnr2.1524 (PMC9327662; doi:10.1002/cnr2.1524)
Supplement: Supplementary file 5 — Supplementary Figure S4 BTK serum expression profile of pre‐treatment and on‐treatment samples grouped by patients with A) early and B) late progression. For missing samples, median value was used as a substitute. [file CNR2-5-e1524-s002.pdf]

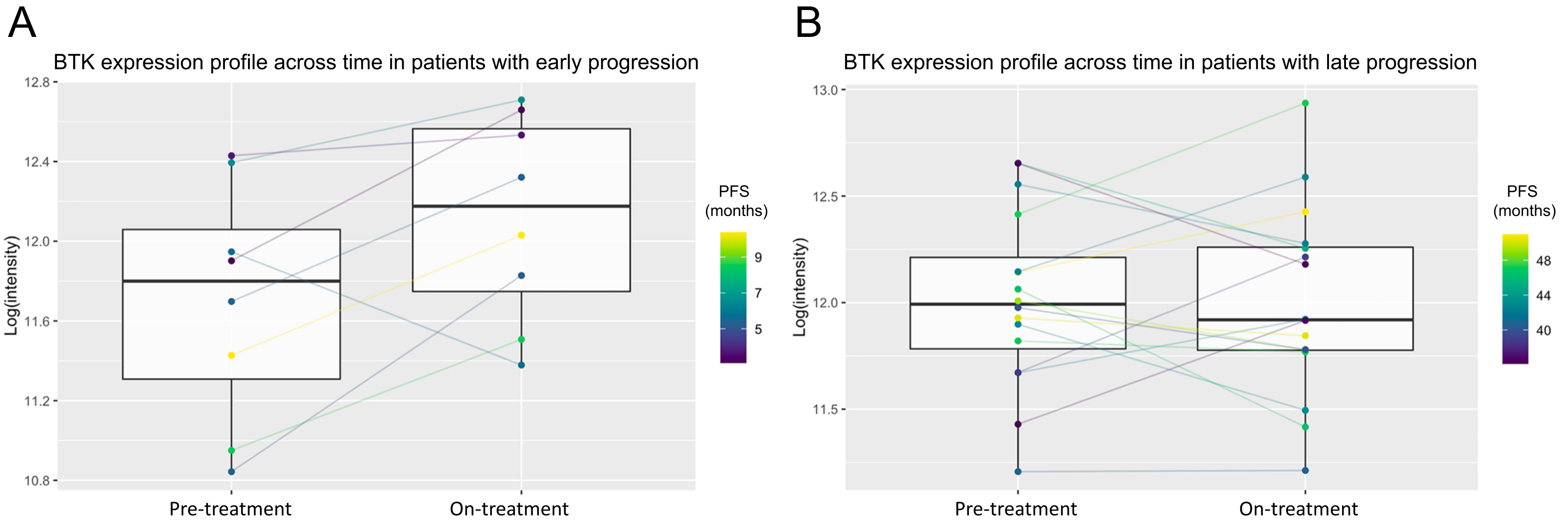
A
BTK expression profile across time in patients with early progression
Log(intensity)
12.8
12.4
12.0
11.6
11.2
10.8
Pre-treatment
On-treatment
PFS (months)
9
7
5
B
BTK expression profile across time in patients with late progression
Log(intensity)
13.0
12.5
12.0
11.5
Pre-treatment
On-treatment
PFS (months)
48
44
40
